# Access of people with pulmonary tuberculosis to government programs: Primary Care professionals’ perceptions

**DOI:** 10.1590/0034-7167-2022-0716

**Published:** 2023-11-13

**Authors:** Hildegard Soares Barrozo de Lima, Vitória Regina Domingues Sodré, Cleide Aparecida Alves Souza, Mirian Domingos Cardoso, Crhistinne Cavalheiro Maymone Gonçalves, Laura Maria Vidal Nogueira, Ivaneide Leal Ataíde Rodrigues, Erlon Gabriel Rego de Andrade, Alexandre Aguiar Pereira, Maria Catarina Salvador da Motta, Maria Helena do Nascimento Souza, Regina Célia Gollner Zeitoune, Ethel Leonor Noia Maciel

**Affiliations:** I Universidade Federal do Rio de Janeiro. Rio de Janeiro, Rio de Janeiro, Brazil; II Universidade Federal de Mato Grosso do Sul. Campo Grande, Mato Grosso do Sul, Brazil; III Universidade de Pernambuco. Recife, Pernambuco, Brazil; IV Universidade do Estado do Pará. Belém, Pará, Brazil; V Universidade Federal do Espírito Santo. Vitória, Espírito Santo, Brazil

**Keywords:** Tuberculosis, Pulmonary, Social Programs, Social Vulnerability, Health Personnel, Primary Health Care, Tuberculosis Pulmonar, Programas Sociales, Vulnerabilidad Social, Personal de Salud, Atención Primaria de Salud, Tuberculose Pulmonar, Programas Sociais, Vulnerabilidade Social, Pessoal de Saúde, Atenção Primária à Saúde

## Abstract

**Objective:**

to analyze Primary Health Care professionals’ perceptions about the access of people with pulmonary tuberculosis to government social support and income transfer programs.

**Methods:**

multicenter/qualitative study, carried out in Family Health Units in four Brazilian capitals: Belém/Pará, Campo Grande/Mato Grosso do Sul, Recife/Pernambuco and Rio de Janeiro/Rio de Janeiro. Fifty-eight professionals participated (social workers, dentists, nurses, pharmacists, physicians and nursing technicians), who provided assistance to people with pulmonary tuberculosis. Individual interviews were conducted, and the content analysis technique was used.

**Results:**

among the participants, 45/77.6% were women and 33/56.9% were between 25 and 40 years old. Two thematic categories were organized, demonstrating the perceptions about the possibilities of access to government programs by people with pulmonary tuberculosis in vulnerable situations and the obstacles inherent to this context.

**Final considerations:**

it is necessary to move forward in improving patient access to social programs.

## RESUMO

**Objetivo:**

analisar as percepções dos profissionais da Atenção Primária à Saúde sobre o acesso de pessoas com tuberculose pulmonar aos programas sociais governamentais de suporte e de transferência de renda.

**Métodos:**

estudo multicêntrico/qualitativo, realizado em Unidades de Saúde da Família de quatro capitais brasileiras: Belém/Pará, Campo Grande/Mato Grosso do Sul, Recife/Pernambuco e Rio de Janeiro/Rio de Janeiro. Participaram 58 profissionais (assistentes sociais, dentistas, enfermeiros, farmacêuticos, médicos e técnicos de enfermagem), que prestavam assistência às pessoas com tuberculose pulmonar. Foram realizadas entrevistas individuais, e utilizou-se a técnica de análise de conteúdo.

**Resultados:**

entre os participantes, 45/77,6% eram mulheres e 33/56,9% tinham entre 25 e 40 anos. Organizaram-se duas categorias temáticas, demonstrando as percepções acerca das possibilidades de acesso aos programas governamentais por pessoas com tuberculose pulmonar em situação de vulnerabilidade e os obstáculos inerentes a esse contexto.

**Considerações finais:**

é necessário avançar na melhoria do acesso dos doentes aos programas sociais.

## RESUMEN

**Objetivo:**

analizar las percepciones de los profesionales de la Atención Primaria de Salud sobre el acceso de las personas con tuberculosis pulmonar a los programas gubernamentales de apoyo social y transferencia de ingresos.

**Métodos:**

estudio multicéntrico/cualitativo, realizado en Unidades de Salud de la Familia en cuatro capitales brasileñas: Belém/Pará, Campo Grande/Mato Grosso do Sul, Recife/Pernambuco y Rio de Janeiro/Rio de Janeiro. Participaron 58 profesionales (trabajadores sociales, odontólogos, enfermeros, farmacéuticos, médicos y técnicos de enfermería) que brindaron asistencia a personas con tuberculosis pulmonar. Se realizaron entrevistas individuales y se utilizó la técnica de análisis de contenido.

**Resultados:**

entre los participantes, 45/77,6% eran mujeres y 33/56,9% tenían entre 25 y 40 años. Se organizaron dos categorías temáticas, demostrando las percepciones sobre las posibilidades de acceso a los programas gubernamentales por parte de las personas con tuberculosis pulmonar en situación de vulnerabilidad y los obstáculos inherentes a este contexto.

**Consideraciones finales:**

es necesario avanzar en mejorar el acceso de los pacientes a los programas sociales.

## INTRODUCTION

Tuberculosis (TB) is a transmissible infectious disease, which in contemporary society still constitutes a relevant public health problem, especially when those affected live in a context of socioeconomic vulnerability^(1)^. This reinforces TB as a condition that demands joint investments, from different actors and governmental instances, for its effective coping^(2, 3, 4)^.

Despite numerous government efforts and on the part of health professionals, there is still a high prevalence of TB at national and international levels. According to the World Health Organization (WHO), in 2021, 6.4 million newly diagnosed cases were officially reported on the global stage. However, it is estimated that 10.6 million people fell ill with TB that year and that the disease caused 1.4 million deaths among people not infected with the human immunodeficiency virus (HIV)^(4)^.

In this context, Brazil appears on two lists that bring together 30 countries with the highest burden of the disease and TB/HIV co-infection^(4)^. Data from the Ministry of Health indicate that, in 2021, 68,271 new cases were registered in the country, with an incidence coefficient of 32.0 cases/100,000 inhabitants. In 2020, 4,543 deaths were reported, with a mortality rate of 2.1 deaths/100,000 inhabitants^(5)^.

Considering the need to promote effective actions that contribute to controlling the disease in Brazil, the Ministry of Health, in 2021, released official recommendations, from 2021 to 2025, which will guide the second phase of the Brazilian National Plan to End TB as a Public Health Problem. This initiative is based on international agreements, such as the Sustainable Development Goals inherent in the 2030 global agenda and the End TB Strategy, declaring goals to reduce the incidence of TB by 90% and deaths from the disease in the country by 95% by 2035. This implies an incidence rate of less than 10 cases/100,000 inhabitants and an annual number of deaths of less than 230^(5, 6)^.

However, it should be noted that, as of 2020, TB control has become an even greater challenge in the world and in Brazil, considering the impacts of the COVID-19 pandemic on reporting cases and deaths from TB as well as in the planning and management of resources to face it within the scope of health services and systems^(5, 7, 8)^. In view of this, in 2020, Brazil and 15 other countries showed a 93% reduction in reports regarding the disease^(9)^.

Among the conditions and determinants of this condition in the population, it is noted that socioeconomic factors have a great influence on the therapeutic process and on the increase in dropout rates, since the increase in the number of cases and deaths from TB is directly associated with conditions of poverty, such as low income, low education, inadequate housing conditions and lack or lack of basic sanitation^(10, 11, 12)^.

The provision of government social support and income transfer programs can contribute in the economic context to improve quality of life and, consequently, to cure TB. In this regard, among the current programs in Brazil, the following stand out: Family Allowance Program (*Programa Bolsa Família*); Benefit of Continued Provision (BPC - *Benefício de Prestação Continuada*); Social Electricity Tariff (*Tarifa Social de Energia Elétrica*); My Home/My Life Program (*Programa Minha Casa/Minha Vida*); Older Adult Card (*Carteira do Idoso*); Retirement for Low-Income People (*Aposentadoria para Pessoas de Baixa Renda*); and Popular Phone (*Telefone Popular*)^(13)^. Although these and other benefits are not specific to people with chronic diseases, the fact of knowing the possibilities of supply allows understanding government initiatives of social protection that can be implemented and strengthened in the care for people with pulmonary TB^(14, 15)^.

In this context, it is known that a significant portion of care for people with TB occurs within the scope of Primary Health Care (PHC), through the resources and social mechanisms available therein, which allow greater approximation between different human groups and health professionals, as these professionals interact and create possibilities to strengthen bonds of trust and communication with human groups^(16, 17)^.

A care model inherent to primary care, Family Health Strategy (FHS) operates its actions in greater proximity to the community^(18, 19)^, scenario in which professionals can understand the conditions of access of people with TB to social programs and contribute to expanding this access, according to their attributions and the limits of their socio-political governance^(14, 15)^.

In the field of health, it is considered opportune to reflect on the concept of access and differentiate it epistemologically from the concept of accessibility. In this study, access is understood as the possibility for users to enter health services to solve their biopsychosocial needs, reason why it is related to the geographical location of the institutions where the services take place, with the availability of days and hours of service and with the possibility of professionals assisting without prior appointment^(20)^.

In turn, resulting from the interaction between aspects of different natures, such as sociocultural, economic, geographic and organizational factors, accessibility implies adjustment between health resource and human group characteristics, when seeking and obtaining care actions. By verifying the conditions of accessibility of a population, it is possible to identify the aspects that facilitate or hinder individual and collective strategies in the search and achievement of these actions^(20)^.

In view of this, it is understood that it is necessary to expand the technical-scientific knowledge about the subject and to make efforts to discuss it and disseminate it in the areas of care, management, teaching and research in health.

## OBJECTIVE

To analyze Primary Health Care professionals’ perceptions about the access of people with pulmonary tuberculosis to government social support and income transfer programs.

## METHODS

### Ethical aspects

According to Resolution 466/2012 of the Brazilian National Health Council^(21)^, the study was approved by the Research Ethics Committee of the *Universidade Federal do Espírito Santo*, the Research Ethics Committee of the *Escola de Enfermagem Anna Nery/Universidade Federal do Rio de Janeiro* and the Municipal Health Department of Rio de Janeiro as well as the Municipal Health Departments of Belém, Campo Grande and Recife.

Professionals had previous contact with the researchers, and read and signed the Informed Consent Form (ICF), expressing their agreement to participate and their awareness of the objectives, risks and benefits. There was no refusal to participate in the study. In order to guarantee the secrecy of identities, the excerpts from the testimonies were coded with the following elements: letter P, for “participant”, followed by an Arabic number corresponding to the order of the interviews, hyphen, abbreviation of the professional category (Sow = social worker; Dent = dentist; Nur = nurse; Pha = pharmacist; Phy = physician; Nur Tech = nursing technician), hyphen and official acronym of the Brazilian state referring to the place of study (MS, PA, PE and RJ).

### Study design

This is a qualitative multicenter study, the first stage of the research project entitled “*Estudo Longitudinal dos Impactos do Suporte Social Indicadores Operacionais da Tuberculose (ELISIOSTB)*”. The writing was guided by the COnsolidated criteria for REporting Qualitative research (COREQ) recommendations^(22)^.

### Study setting and data source

The research was carried out in Family Health Units (FHU) in four Brazilian capitals: Belém (PA), Campo Grande (MS), Recife (PE) and Rio de Janeiro (RJ). These locations were chosen because they are the preferential gateway to access health services. The municipalities were chosen due to the high incidence of TB, according to data published by the Ministry of Health in a special issue of the TB Epidemiological Bulletin^(5)^, which indicate the following incidence rates (number of cases/100,000 inhabitants) for 2021: Belém, 67.5; Campo Grande, 35.8; Recife, 89.8; and Rio de Janeiro, 92.5^(5)^.

A total of 58 professionals participated (five from Belém, 22 from Campo Grande, three from Recife and 28 from Rio de Janeiro), who worked in partner health units as supervised internship fields for undergraduate nursing students from the universities involved in the project. Professionals who provided assistance to people with pulmonary TB in health services during the data collection period were included, regardless of their job tenure. Those who were on vacation or on leave at that time were excluded.

Professionals were selected for convenience, with voluntary indication by their peers as they participated in the research. Considering the period foreseen to collect the data and, due to this selection strategy, there was no parity between the numbers of participants in the municipalities studied, without prejudice to developing the research.

### Data collection and organization

As a collection technique, semi-structured individual interviews were chosen, carried out from July 2019 to February 2020. They were conducted by the researchers and fellows linked to the project: master’s students and previously trained nursing students.

A script was used, previously prepared by the project team, containing six questions to characterize participants’ sociodemo-graphic and occupational profile and two guiding questions to know the perceptions and identify the object of study. The profile questions investigated six variables: sex; age; profession; tenure at the health unit; length working in caring for people with TB; and participation in training activities for case management. The guiding questions were: what types of government social support and income transfer programs can be offered to people with pulmonary TB? How do people with pulmonary TB access government social support and income transfer programs?

To guide qualitative data collection, in addition to the guiding questions, participants were presented with a list of governmental social support and income transfer actions or programs, previously prepared by the team, in order to help them answer. In line with the collection technique, the guiding questions were deployed in order to encourage participants to discuss the topic in greater depth. These developments occurred through dialogue with participants, as the researchers realized the need to know, deepen or clarify relevant aspects in the reports.

Whenever necessary, questions were added to complement the reports and better understand the perceptions such as: in the health unit where you work, do people with pulmonary TB have access to the social programs mentioned by you? In this context, how do you access them? What difficulties or obstacles do they face in obtaining the social benefits to which they are entitled?

The interviews lasted from 20 to 30 minutes and were held in reserved places on the premises of the health units to ensure comfort and privacy for participants. In order to identify all the nuances of the testimonies, the interviews were recorded in MP3 format, with participants’ formal authorization when signing the ICF. The recordings proved sufficient to capture the nuances, which is why techniques were not employed to manually record any qualitative data. Respecting data confidentiality, it was decided to store the audio files on a personal computer with a password, whose access was exclusive to the project team.

### Data analysis

The Statistical Package for the Social Sciences (SPSS), version 21.0, was used to process participants’ profile data, in order to present absolute values and respective percentages.

The interviews were transcribed and analyzed by the team, using the thematic content analysis technique^(23)^, following its three stages: pre-analysis – attentive and detailed reading of the testimonies; material exploration – grouping speech excerpts into recording units to form thematic categories with complementary themes, which would express the qualitative data completeness; and treatment of results – inferential and interpretative synthesis of these categories, supported by evidence from the updated scientific literature on the subject, aiming to present robust and pertinent discussions^(23)^.

After transcription, the team kept the audio files carefully stored in order to consult them to clarify possible doubts regarding the text *corpus* content. However, they will be destroyed five years after the data collection period, reaffirming the researchers’ ethical and technical-scientific commitment.

## RESULTS

Among the 58 professionals who provided assistance to people with pulmonary TB, 45 (77.6%) were female and 33 (56.9%) stated that they were between 25 and 40 years old. With regard to professions, two (3.4%) were social workers; three (5.2%), dentists; 21 (36.2%), nurses; four (6.9%), pharmacists; 18 (31.0%), physicians; and 10 (17.2%), nursing technicians.

Tenure at the health unit ranged from less than one year to 22 years, with 30 (51.7%) working for five years. The length working in caring for people with TB also ranged from less than one year to 22 years, a context in which 32 (55.2%) reported job tenure of less than five years. As for training, 36 (62.1%) declared participation in a training course for the management of people with TB.

By analyzing professionals’ perceptions about the access of people with pulmonary TB to government social support and income transfer programs, two thematic categories emerged: *Possibilities of access to government programs by vulnerable people with pulmonary tuberculosis*; *Obstacles to accessing government programs by people with pulmonary tuberculosi*s. These categories considered and valued all participants’ perceptions. However, striving for clarity and organization of results, we chose to highlight the most emblematic excerpts, corresponding to 10 participants, as described below.

Although there are technical-scientific and practical specificities between the professions represented here, it is worth noting that this study did not seek to associate perceptions with these characteristics, which is why, *a priori*, the excerpts do not reflect the particularities of each profession in the set of qualitative data.

### Possibilities of access to government programs by vulnerable people with pulmonary tuberculosis

In this category, a set of possibilities for people undergoing TB treatment to access government benefits was shown. During the interviews, from the visualization of a list of actions or governmental social support and income transfer programs, participants reported that people with pulmonary TB, depending on the need, can have access to benefits like any other user in a situation of social vulnerability:

*The person with TB is in a situation of vulnerability. So, these programs* [Family Allowance, BPC, Transportation vouchers, among others] *are also for this vulnerable population.* (P3_Phy_RJ)

*The social programs that people with TB have access to today are the same as any other low-income population. The fact that the person has TB or not is not a factor that interferes with access to programs.* (P15_Phy_RJ)

*If they are a TB patient and has a low income, I refer them to a CRAS* [Social Assistance Reference Center]*.* (P2_Sow_PA)

*They* [patients] *are referred to the CRAS and many of them already have a bond. Due to their social condition, they already have access to the Family Allowance.* (P1_Nur_PE)

In this perspective, given the limitations and needs imposed by the illness, professionals referred people with TB to the Social Assistance Reference Center (CRAS - *Centro de Referência de Assistência Social*) so that they could have access to the Family Allowance Program, transportation vouchers, among other benefits. Thus, the possibility of access to government benefits was affirmed by all professionals, regardless of the peculiarities of the Brazilian regions where the study was carried out.

### Obstacles to accessing government programs by people with pulmonary tuberculosis

Through this category, it was demonstrated that there are difficulties and obstacles for people with pulmonary TB to obtain the benefits of government social support and income transfer programs. Among them, reduction or discontinuation of programs or benefits, lack of documents by users, limitations of accessibility to services, bureaucratic and political issues, long waiting time to release these benefits and unfavorable social conditions of users are mentioned:

[...] *the INSS issue* [referring to the bureaucratic procedures of the Brazilian National Institute of Social Security] *is very complicated. The insertion of people is extremely difficult, they ask for a series of documents that the person does not have. And for our patients who are homeless, it gets much more complicated. Patients are not having access to benefits and this makes treatment evolution very difficult.* (P1_Nur_RJ)

*Access is not easy. At the moment, I don’t have any patients who are receiving benefits.* (P3_Nur_PE)

*Nowadays, unfortunately, we are not having this kind of* benefit [referring to government social programs] *for patients, I don’t know exactly why.* (P17_Nur_MS)

*Social programs exist, but to have access you have to have a series of documents, a series of bureaucracies, which make people give up.* (P3_Nur Tech_PA)

[...] *access is not easy* [...]*. For example, if they* [patients] *arrive here, I forward them to the CRAS* [...], *I forward them because I have to advise on the report. They cannot go to the CRAS, request an assistance benefit, if they do not bring a report* [...]*. It’s a rush from door to door, and he goes knocking, knocking. And there comes a time when he even gives up because he is already getting better, the fever is already going away, the infection is decreasing, the TB crosses* [which indicate the severity of the infection] *are decreasing, and then he says: “I’m already tired* [...]*, I’m already getting better, I won’t look for it* [...]*”. But it’s hard, the door isn’t open, it’s not.* (P2_Sow_PA)

[...] *we forward it to the CRAS for a registration, which will generate a NIS* [Social Identification Number] *for that patient, who will see which social program he fits.* (P18_Sow_MS)

[...] *it is valid for transportation, if the person lives far away, but the assistance is only available to people in the* region [explaining that, for operational reasons, access to this benefit often has limitations for people who live in distant locations, even that are attended by the health unit]*.* [...] *I am not aware of the periodicity* [of access to this benefit]*, but I know that they* [patients] *earn.* (P12_Phar_MS)

It was identified that access to government social programs is strongly permeated by obstacles of an administrative and operational nature, mainly materialized by bureaucratic work processes. This reality was demonstrated throughout the study scenario, regardless of the sociodemographic and epidemiological characteristics of the Brazilian regions, since the obstacles were reported by all participants and highlighted as limiting elements, whose overcoming is necessary and constitutes a collective challenge.

## DISCUSSION

The study identified aspects that evidenced PHC professionals’ perceptions regarding the access of people with pulmonary TB to government social support and income transfer programs.

In this regard, it is highlighted that cash transfer programs are innovative social protection strategies. They consist of granting financial benefits to individuals and families in a situation of socioeconomic vulnerability and aim to overcome poverty by strengthening the financial, physical and human capital of these individuals^(13, 24)^.

One of the most relevant risk factors in the social context of people with TB is their vulnerability, which is why it is also necessary to consider social inequality, with a view to establishing and monitoring adequate treatment^(25)^. Therefore, in addition to diagnosis, drug treatment and regular consultations, conditions that must be offered in health care networks, it is clear that people in this situation of vulnerability need to be covered with social benefits or income transfer, essential for continuity of treatment and for the process of curing the disease.

In professionals’ perceptions, regardless of TB diagnosis, people in vulnerable situations are, or should be, referred to a CRAS, for the adequate care for their support needs. Although most participants demonstrated knowledge of the existence of programs such as Family Allowance Program, BPC, My Home/My Life and transportation vouchers, they still reported low access for TB patients due to difficulties in referral and excessive bureaucratic procedures. Moreover, there are sometimes gaps in professionals’ knowledge of available government support programs, characterizing itself as an obstacle to the more conscious and safer performance of these professionals, considering that their actions, intrinsic to PHC, occur in scenarios with significant demands of a social and economic nature^(14)^.

Professionals’ knowledge about these social devices is fundamental to guide and clarify users, allowing them to have access and enjoy the rights that are inherent to them regarding the resources of government programs and income distribution. This knowledge gains greater relevance because, for the most part, people with TB are in the condition of socioeconomic vulnerability and because it helps to face obstacles, in order to provide disease treatment and cure^(15)^.

The Ministry of Health and the Ministry of Citizenship in Brazil have made efforts to implement government policies aimed at social protection and considering that TB is a disease that mainly affects low-income populations. The result of these initiatives is contained in the publication of Joint Operational Instruction 1 of September 26, 2019, which specifies guidelines on the performance of the Unified Social Assistance System (SUAS - *Sistema Único de Assistência Social*) together with the Unified Health System (SUS - *Sistema Único de Saúde*) for combating TB^(26)^.

Thus, the importance of joint activities between public health and social assistance policies is evident, with a focus on individual and collective vulnerabilities, in order to achieve favorable outcomes for neglected diseases such as TB. It is also emphasized the need for a therapeutic project that responds assertively to the needs of people affected by the disease, especially if they have financial difficulties, characteristics of the poverty condition in which they live^(10, 27)^.

Faced with the singularities of a person undergoing treatment and the biopsychosocial needs that arise from their clinical condition, the Singular Therapeutic Plan (PTS - *Plano Terapêutico Singular*) is an important tool for the multidisciplinary team to reach an agreement, with users and their support network, on ways of caring focused on a human person’s comprehensiveness, with the aim of consolidating links with health services and strengthening the therapeutic process, especially in cases with a higher degree of complexity. It can be carried out at any level of care, allowing professionals to share knowledge and experiences to clarify doubts about the disease and the proposed and implemented actions^(28)^.

To be effective in TB control, this plan includes a situational diagnosis of users’ life and health conditions; the definition of pertinent and achievable goals; the establishment of co-responsibility among team members, from which a reference professional is defined, who will coordinate the PTS, usually the one with whom users have the greatest affinity; and reassessment, conducted by the plan coordinator and operationalized through meetings with the different actors so that expectations, goals, deadlines, results and other aspects are reviewed, and measures are taken^(28)^.

In this scenario, access to social income transfer programs implies a positive impact on TB treatment outcomes for the poorest population, indicating the need to address the illness in a comprehensive and effective way beyond the disease^(10, 13, 24, 25, 29)^. Corroborating this statement, a cohort study carried out in Salvador (BA), with 216 people affected by TB, analyzed associations between treatment outcome, sociodemographic variables and social benefits, demonstrating that 172 (79.6%) were cured and the highest proportion of this outcome occurred among people who received governmental and non-governmental benefits (n=19/21; 90.5%) or only direct benefits (n=124/152; 81.6%), i.e., monetary resources transferred directly to beneficiaries^(24)^.

A systematic review with meta-analysis of studies carried out in low- or middle-income countries or with a high burden of TB, such as Brazil, investigated the effects of social protection strategies on disease treatment outcomes. The qualitative synthesis brought together 25 studies, of which nine were used in the meta-analysis because they were randomized and controlled studies, with a total number of participants of 1,687. Among other aspects, it was identified that social protection strategies were associated with curing patients (RR=1.11; 95%CI: 1.01-1.22) and reducing the risk of treatment abandonment (RR=0.63; 95%CI: 0.45-0.89)^(10)^.

Therefore, it is imperative that public policies are based on principles that address the real needs of people undergoing treatment. However, it is not enough to guarantee access and priority service, as it is also important to safeguard each person’s uniqueness and citizenship rights, regardless of their risk level and/or social condition^(30)^.

Treatment success should not be considered only from the point of view of medication compliance, but as a dynamic and integrated set of actions, in which health professionals approach individuals holistically, involving their family context and offering support and referral to other services, when necessary^(7, 12, 30)^.

It was found that, after referring users to a CRAS, professionals found it difficult to identify whether they received support from government programs, revealing the weakness in counter-referral to the PHC unit. Corroborating this finding, a study carried out in Recife (PE) showed the lack of articulation and counter-reference between the PHC unit and other services of the Health Care Network that provided care to people with TB^(31)^. It should be noted that it is necessary to know the referral and counter-referral processes, management tools that the SUS presents for its consolidation^(32)^.

As obstacles to accessing social protection benefits, participants mentioned situations such as lengthy bureaucratic processes, lack of user documents and delay in receiving benefits approved by the government. This fact reflects the long way to go for social programs to be effectively implemented at the national level, thus constituting mechanisms to help combat TB in Brazil^(11, 12, 29)^.

Furthermore, it is worth emphasizing that people with TB may face barriers inherent to health units’ opening hours, lack or lack of medication, inadequate control of contacts, late disease diagnosis and repeated trips to health services to obtain a diagnosis^(30, 33)^.

Confirming the reflection on the need to implement social programs and thus mitigate obstacles to reducing TB figures, a study on the social determinants and targets for the disease in the Americas reinforces the need to expand health promotion and disease prevention measures, reducing access barriers to diagnosis and treatment and strengthening initiatives that aim to address the social determinants related to the disease^(34)^.

Barriers to access social benefits and income transfer can negatively influence disease control, because, in health professionals’ care practice, social inequalities and accelerated population growth, added to determining factors such as age, education, comorbidities, consumption of alcohol and other drugs, food and nutritional insecurity, precarious home conditions and difficulties in accessing and accessibility to health services in the Brazilian territory, impact the infectious agent transmission chain, disease progression and, consequently, treatment outcomes^(1, 7, 25, 35)^.

The challenges of this scenario, aggravated by access barriers in the Brazilian territory, can lead to interruptions in TB treatment or even therapeutic abandonment among vulnerable users, since the social determinants, linked to their living conditions and health, limit the possibilities that these people have to follow the therapeutic plan^(15)^. In view of this, the scientific literature has implications for users’ lives and the health system, considering the possibility of worsening their clinical condition, such as resistant or multidrug-resistant mycobacterial infections and progression to conditions that require hospitalization, prolonging treatment duration and increasing public health costs^(28, 36, 37)^.

Among the social support programs, it should be noted that the Family Allowance Program has positive repercussions on the daily lives of many individuals and groups who live in extreme poverty, above all because it is, in a significant number of cases, the only safe source of family income to support survival^(24, 38)^.

With regard to TB control, it is essential to train and raise awareness of professionals working in PHC to learn about users’ reality, especially their socioeconomic demands. Furthermore, it is necessary that they contribute to meeting them, in accordance with their governance and the operational limits of the positions they occupy in health services, and help to strengthen users’ support networks during the stages of treatment^(39, 40)^. To bring the relationship closer to users, home visits are an important resource, from which professionals can better understand life and health processes as well as the demands that arise from them, in order to plan actions and intervene safely and effectiveness^(41)^.

There are several strategies that health units and their multidisciplinary teams should adopt to help patients access social benefits, among which are: know the vulnerability conditions of those affected; verify if they are registered in *CadÚnico* and, if they receive other benefits, stating this information in the medical record; properly fill out the TB reporting form in the Notifiable Diseases Information System (SINAN - *Sistema de Informação de Agravos de Notificação*), paying attention to the variable referring to the Family Allowance Program; create partnerships with institutions/organizations that provide assistance to vulnerable people in the territory to organize flows that allow forwarding and accompanying them in a shared way; actively seek out and investigate respiratory symptoms; carry out health education activities about TB and the benefits to which patients are entitled. From this perspective, it is essential that health and social assistance professionals act in an integrated and collaborative manner^(14)^.

Due to its peculiar socio-historical trajectory, TB is still a disease strongly associated with fears, stigmas and prejudices, materialized in the way people and social groups understand the disease and act towards those affected^(42, 43)^. Despite the widely available technical-scientific knowledge about TB, it is known that many negative social representations are still shared by health professionals and are crystallized in their daily lives, expressing themselves through words, attitudes and behaviors that disqualify those affected and tend to distance them from health services, compromising the therapeutic process^(42, 44)^.

In view of this scenario, in order to collaborate in coping with TB and its biopsychosocial repercussions, it is also necessary for professionals to rethink the language with which they communicate and share information about the disease among themselves and with the population, in order to review and abandon the use of terms and expressions that strengthen stigmas and prejudices. Thus, it is understood that it is possible to mobilize thoughts and actions that empower those affected in self-care and promote their role in health services^(45)^.

### Study limitations

The limitations of this study are related to its development only in PHC health units, located in specific municipalities, reflecting the socio-cultural, political-economic and operational realities of these scenarios. From this perspective, it is understood that, at least partially, this fact may limit the generalization of results.

However, as it was carried out in four capitals in different Brazilian regions, the research increased knowledge about the factors involved in the access of people with TB to government social support and income transfer programs during the phase in which they live with the disease and deal with the biopsychosocial demands that arise from it.

Considering the breadth and scientific and social relevance of the topic, it is understood that other studies are necessary, in order to know, interpret and discuss aspects not investigated here, which can add information that subsidize the global understanding of the topic.

### Contributions to nursing, health, or public policy

In general, the study contributes towards making it possible to deepen and produce new knowledge on the subject, from the perspective of professionals who care for people with pulmonary TB, pointing out and discussing the importance of resource availability, public policy improvement, user access and accessibility to these devices.

In the fields of nursing and health, it is noteworthy that the study contributes with aspects that can support care planning, execution and assessment and other actions that help to face obstacles to access governmental social programs and mitigate their biopsychosocial repercussions in individuals’ and groups’ lives.

## FINAL CONSIDERATIONS

The results of this study made it possible to identify that, from PHC professionals’ perceptions, people with pulmonary TB can have access to benefits from government social support and income transfer programs, like anyone in a vulnerable situation, depending on their needs. From this perspective, they highlighted several obstacles to gaining access.

Considering the reflections brought about by the study, it is indicated the need for the various social actors, such as public authorities, managers, providers and users of health actions and services, to review ways of thinking and acting in the context of care for people with pulmonary TB. This must be done with the aim of promoting and/or favoring conditions that improve access to social programs, assistance to people undergoing treatment and TB control and monitoring actions as well as reduce the number of cases of therapeutic abandonment, providing the cure.
